# Harmful alcohol use and frequent use of marijuana among lifetime problem gamblers and the prevalence of cross-addictive behaviour among Greenland Inuit: evidence from the cross-sectional Inuit health in transition Greenland survey 2006–2010

**DOI:** 10.3402/ijch.v72i0.19551

**Published:** 2013-03-15

**Authors:** Christina Viskum Lytken Larsen, Tine Curtis, Peter Bjerregaard

**Affiliations:** 1 National Institute of Public Health, University of Southern Denmark, Denmark; 2 Local Government Denmark, Denmark

**Keywords:** problem gambling, indigenous health, social pathologies, addictive behaviour, Inuit

## Abstract

**Background and objectives:**

Public health research has pointed to alcohol and substance abuse as the most significant public health challenges in Greenland with the negative impact on families and communities that entail, but few studies have investigated the role of problem gambling as addictive behaviour among Inuit. The objectives of the present study were to investigate (a) the association between lifetime problem gambling and harmful alcohol use as well as frequent use of marijuana and (b) the prevalence of cross-addictive behaviour among Greenland Inuit.

**Design:**

A representative cross-sectional study among Greenland Inuit (n=2,189). Data was collected among adults (18+) in 8 towns and 13 villages in Greenland from 2006–2010. Lifetime problem gambling, harmful alcohol use and frequent use of marijuana were measured through a self-administered questionnaire.

**Results:**

The odds ratio for harmful alcohol use and frequent use of marijuana was significantly higher among lifetime problem gamblers compared to non-problem gamblers/non-gamblers. One or more addictive behaviours were present among more than half of the men (53%) and one third of the women (37%), and the co-occurrence of lifetime problem gambling with either harmful alcohol use, frequent use of marijuana or both was found among 12.2% of men and 3.7% of women. The prevalence of one or more addictive behaviours was 44% in households with children.

**Conclusions:**

For lifetime problem gamblers, the gambling problems were more often than not combined with harmful alcohol use, frequent use of marijuana or both – especially among men. The high prevalence of addictive behaviours in households with children indicates that many families are presently affected negatively by alcohol, gambling and marijuana. This suggests that pathological gambling should be included systematically in future public health strategies, treatment programs and interventions in Greenland.

Public health research has pointed to alcohol and substance abuse as the most significant public health challenge in Greenland. The negative consequences of especially alcohol problems reach far into the lives of communities, families and children. It is well known that use of alcohol and marijuana is often combined, but few studies have investigated how gambling problems might be included in a pattern of cross-addictive behaviours among Greenland Inuit.

In recent years, pathological gambling has become an issue of increasing concern in Greenland (1). For the majority, gambling is a harmless activity, but for some gambling becomes a disorder with a negative impact on their personal lives, families and the communities they live in (2). Little research has been conducted in this area among Greenland Inuit, which might explain why public health programs and intervention strategies have not yet taken pathological gambling into account. In the Inuit Health in Transition Greenland survey 2006–2010, we found the lifetime prevalence for problem gambling to be 9% among women and 16% among men, and the gambling patterns reflected a mix of modern games popular in contemporary Scandinavia and games found to be traditionally popular among Inuit in Nunavik and First Nations (3). The high prevalence of lifetime problem gambling reflects the increase in social pathologies Greenland has experienced during the past 60 years. The rapid social transition Greenland Inuit have gone through has been accompanied by a high prevalence of addictive behaviours (4).

Although the average alcohol intake has decreased since the 1980s, where the alcohol consumption was at its highest (5), a drinking pattern dominated by binge drinking persists as a major cause of social and health problems in Greenland. Today, Greenland is still struggling with the negative consequences for the generations who grew up with alcohol problems in their childhood home such as suicidal behaviour and a high prevalence of violence and sexual abuse (6,7), and, in recent years, increased political attention has been given to improving the lives of children and families. The use of hard drugs has never been a problem in Greenland. Given the geographical isolation, the police have managed to prevent drugs, other than marijuana, from entering Greenland. The issue of substance abuse among Greenland Inuit this primarily regards the use of marijuana.

Several international studies have found problem gambling to be associated with alcohol and substance use as well as mental disorders, but most of these studies have been conducted in a clinical context among pathological gamblers in treatment, and their findings cannot easily be generalised to problem gamblers in the general population. A recent review evaluated the prevalence of comorbid disorders among problem and pathological gamblers in population surveys and pointed to an elevated prevalence of both substance and alcohol use disorders among problem and pathological gamblers compared to non-problem gamblers across studies (8). However, none of these studies had been conducted among indigenous populations, where the prevalence of addictive behaviours is high.

The objectives of the present study were, thus, to investigate (a) the association between lifetime problem gambling and harmful alcohol use as well as frequent use of marijuana and (b) the prevalence of cross-addictive behaviour among Greenland Inuit. We hypothesised that lifetime problem gambling was associated with a higher prevalence of harmful alcohol use and frequent use of marijuana, compared to the prevalence among non-problem gamblers and those who never gambled.

## Material and methods

### Data and sample size

Data was derived from the cross-sectional Inuit Health in Transition Greenland Survey 2005–2010. The study was a population-based general health study among adults (18+). A full methodological report of the study is published elsewhere (9). Greenland was divided into 12 strata based on geography and community size. From each region, a number of towns and villages were selected for the study. A random sample was drawn from the central population register to obtain around 300 participants from the towns included. In the selected villages, everyone was invited to participate. Data were collected by an interviewer-based questionnaire combined with a self-administered questionnaire containing the more sensitive and personal questions, which were believed not to be suited for the face-to-face interview. The questions regarding problem gambling, alcohol use and use of marijuana were all included in the self-administered questionnaire. Questions about gambling were included in the self-administered questionnaire from 2006 and onwards. The 2006–2010 sample included 3,893 Greenland Inuit. A total of 2,451 persons participated in the general survey (63%), and 2,189 persons filled out the self-administered questionnaire (56%). The analyses were based on these 2,189 participants. In total, 2,012 of the 2,189 participants (92%) answered at least one question about gambling.

Despite Greenland's large geographical size, its total population is only about 57,000, of whom 90% are ethnic Greenlanders (Inuit). Genetically, Greenlanders are Inuit (Eskimos) with a mixture of European, mainly Scandinavian, genes. They are closely related to the Inuit/Iñupiat in Canada and Alaska and, somewhat more distantly, to the Yupiit of Alaska and Siberia (10).

### Lifetime problem gambling, harmful alcohol use and frequent use of marijuana

The questions about pathological gambling in this study were a small part of a larger health survey, which forced us to use a short screen. Problem gambling was measured using the Lie/Bet Questionnaire (see Table II) originally suggested by Johnson and Hammer (11) and later validated by both Johnson and Hammer (12) and Götestam et al. (13). The Lie/Bet Questionnaire is a two-question version of the 10 DSM-IV criteria for pathological gambling (2). According to DSM-IV, a positive response to 5 or more of the 10 criteria is characterised as pathological gambling, while three to four positive answers are defined as problem gambling. The lie/bet screen has been found valid to identify lifetime problem gamblers, defined as those who responded positively to 5 or more of the 10 DSM-IV criteria in a combined group with those who responded positively to only three to four of these criteria (13). The short screen cannot distinguish between pathological and problem gambling. Respondents were asked whether or not they had lied to friends and family about their gambling activities, and whether or not they had felt a need to increase bets in the past year or previously in life. Positive answers for the past year and previously were combined for a prevalence of lifetime problem gambling, that is problem gambling that had occurred at some point in life. Analyses are based on lifetime gamblers as opposed to past year gamblers in order include enough individuals to be able to analyse subgroups in the sample. Non-problem gamblers and those who never gamble are collapsed into one group in the analyses. When mentioned in the text, they are called non-problem gamblers, although they include a small group who never gamble.

**Fig. 1:**
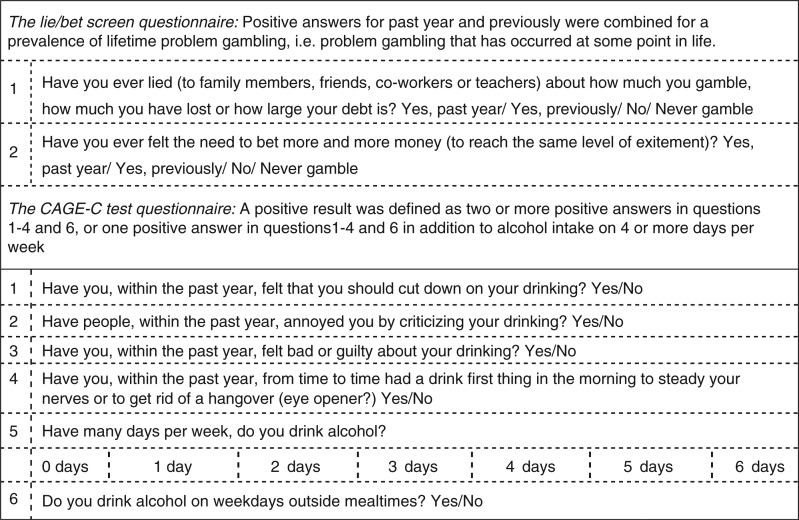
Questionnaires for the Lie/Bet screen and the CAGE-C test used in the Inuit Health in Transition Greenland Survey 2006–2010.

Harmful alcohol use was measured by the modified CAGE-test: CAGE-C. It is a simple screening tool suited for identifying alcohol problems in populations with a high prevalence of at-risk drinkers. The original CAGE test was based on a 4-item questionnaire and measured harmful alcohol use in a lifetime perspective (14). However, the validity of the original questionnaire outside a clinical context has been questioned (15), and the sensitivity of the test has been criticised in several studies (16,17). The 6-item questionnaire CAGE-C was suggested by Zierau et al. in 2005 (18) and validated against a diagnostic interview based on ICD-10 (19) and DSM-III R (20) criteria. The questionnaire (Fig. 1) has been used to assess harmful alcohol intake among Greenland Inuit in an earlier study (21). The modified CAGE-test was included in the self-administered questionnaire. It measures harmful alcohol use in a past year perspective and includes a question regarding the number of days per week of alcohol use and a question concerning alcohol intake on weekdays outside meals. CAGE positives were defined by a positive answer in two or more of questions 1–4 and 6, or one positive answer in question 1–4 and 6, in addition to alcohol intake on 4 or more days per week.

Frequent use of marijuana in the past year was measured through two questions in the self-administered questionnaire. Participants were asked if they had ever smoked marijuana. Those who answered “yes, once or a few times” or “yes, several times” were asked how often they had smoked marijuana in the past year. Those who had tried smoking marijuana and additionally answered that they had smoked at least one to three times a month in the past year were categorised as past-year frequent users of marijuana. Those who had never tried to smoke and those who had smoked less than once a month in the past year were categorised together as non-frequent users/non-users.

### Socio-demographic variables

Age, place of residence and formal education were included as socio-demographic variables. To ensure enough observations, age groups were collapsed into two groups of 18–34 years and 35+ years. Formal education was determined from questions about highest school education attained and further vocational or academic education, and recoded into primary school/high school only, short vocational education (less than 3 years), and longer vocational/academic education. Information on the socio-demographic variables was obtained through the interviewer-based questionnaire.

Place of residence was divided between the capital Nuuk, towns and villages. Greenland has a total of 80 communities all located on the coast. A town is defined historically as the largest community in each of 17 districts. In 2010, the population of Nuuk was 15,469, the population of the towns varied between 469 and 5,460, while that of the villages varied from less than 10 to around 550. In the towns are located district school(s), a health centre or hospital, church, district administration and main shops. These institutions are absent or present to a much smaller extent in the villages. The isolated populations in the villages and smaller towns face the largest challenges with limited job and recreational opportunities. In contrast to these, the capital, Nuuk, with its more than 15,000 inhabitants is a modern town reflecting contemporary Scandinavian lifestyle with a wide range of educational, occupational and recreational possibilities (22).

### Alcohol problems in the childhood home

Traumatic events during childhood have been found to be associated with problem gambling in previous studies (23). Therefore, logistic regressions were adjusted for alcohol problems in childhood in the analyses of the association between lifetime problem gambling, harmful alcohol use and frequent use of marijuana. Participants were asked through the self-administered questionnaire if there were alcohol problems in their childhood home (never; yes, occasionally; yes, often).

### Statistical analyses

Differences between problem gamblers and non-problem gamblers regarding harmful alcohol use and frequent use of marijuana according to gender, age group and place of residence were tested with chi-square test. Prevalence estimates were weighted for regional and age differences in the sample. Analyses were done by logistic regression stratified for gender. Results are reported as odds ratios (OR) with a 95% confidence interval (CI). Both adjusted and unadjusted ORs are reported.

### Ethical review

The study was ethically approved by the Commission for Scientific Research in Greenland. Participants gave their written consent after being informed about the study orally and in writing.

## Results

### Socio-demographic variation in response

The variables included in the study are displayed in Table I. Initially the response rate for the key variables included was analysed for associations with socio-demographic variables (results not tabulated). Response to questions regarding problem gambling was associated with gender (p<0.0001), formal education (p<0.0001) and age group (p<0.0001). Response to questions about harmful alcohol use was associated with gender (p=0.012), formal education (p=0.004), age group (p<0.0001) and place of residence (p<0.0001). Response to questions about use of marijuana was associated with gender (p<0.0001), formal education (p<0.0001), age group (p<0.0001) and place of residence (p<0.0001). In general, the odds ratio for having answered the questions was higher among men than women, and among inhabitants from the capital Nuuk compared to inhabitants from towns and villages. The odds ratio decreased with age and increased with level of education.

**Table I:** Overview of variables included. Frequency, valid percent, N, missing data and socio-demographic associations in response rates for the variables regarding problem gambling, alcohol use and marijuana

	Frequency (%)	N	Missing
Total		2,189	0
Men	983 (44.9)		
Women	1,206 (55.1)		
Age groups		2,189	0
18–24 years	278 (12.7)		
25–34 years	385 (17.6)		
35–59 years	1,273 (58.2)		
60+	253 (11.6)		
Place of residence		2,189	0
Nuuk	419 (19.1)		
Town	1,110 (50.7)		
Village	660 (30.2)		
Lifetime problem gambling (Lie/bet screen)		1,542	647
Yes	196 (12.7)		
No	1,346 (87.3)		
Harmful alcohol use past year (CAGE-C)		1,599	590
Yes	521 (32.6)		
No	1,078 (67.4)		
Frequent use of marijuana past year		1,833	356
Yes	250 (13.6)		
No	1,583 (86.4)		

Response rates were analysed by logistic regression for associations with gender, age, place of residence and level of education. Greenland Inuit 2006–2010.

### Harmful alcohol use

Lifetime problem gambling was significantly associated with a harmful use of alcohol among men, but not among women (Table II), and the odds ratio for harmful alcohol use was twice as high for male lifetime problem gamblers as for non-problem gamblers (both unadjusted and adjusted). The prevalence of harmful alcohol use among lifetime problem gamblers compared to non-problem gamblers was significantly higher for those aged 35+. Among lifetime problem gamblers, the prevalence of harmful alcohol use increased with age, while decreasing with age among the non-problem gamblers (men and women). Harmful alcohol use was more prevalent among lifetime problem gamblers compared to non-problem gamblers in towns and villages, but not in the capital (men only).

**Table II:** Prevalence of harmful alcohol use and frequent use of marijuana in past year among lifetime problem gamblers compared to non-problem gamblers/non-gamblers according to gender, age and place of residence

	Harmful alcohol use past year (CAGE-C)			Frequent use of marijuana past year		
		
	Lifetime problem gamblers	Non-problem gamblers/non-gamblers	p	Lifetime problem gamblers	Non-problem gamblers/non-gamblers	p
Men						
Odds ratio (95% CI)						
Unadjusted	2.24 (1.44–3.48)	1 (ref.)	**<0.0001**	1.74 (1.07–2.83)	1 (ref.)	**0.027**
Adjusted for age and place of residence	2.26 (1.45–3.54)	1 (ref.)	**<0.0001**	1.73 (1.06–2.85)	1 (ref.)	**0.030**
Adjusted for age, place of residence and alcohol problems in childhood home	2.14 (1.35–3.41)	1 (ref.)	**0.001**	1.56 (0.94–2.59)	1 (ref.)	0.089
Percent (N) *All men*	49.6% (54)	34.4% (178)	**0.001**	40.6% (27)	16.8% (94)	**<0.0001**
Age groups						
18–34 years	34.1% (17)	45.3% (57)	0.207	40.0% (11)	20.4% (30)	**0.007**
35+ years	58.7% (37)	29.1% (121)	**<0.0001**	40.3% (16)	15.2% (41)	**<0.0001**
Place of residence						
Nuuk	40.0% (5)	27.2% (28)	0.303*			
Town	53.3% (34)	36.4% (91)	**0.008**			
Village	73.1% (15)	41.7% (59)	**0.005**			
Women						
Odds ratio (95% CI)						
Unadjusted	1.58 (0.90–2.77)	1 (ref.)	0.115	2.75 (1.44–5.25)	1 (ref.)	**0.002**
Adjusted for age and place of residence	1.59 (0.90–2.81)	1 (ref.)	0.109	2.98 (1.54–5.79)	1 (ref.)	**0.001**
Adjusted for age, place of residence and alcohol problems in childhood home	1.26 (0.70–2.26)	1 (ref.)	0.442	2.69 (1.35–5.34)	1 (ref.)	**0.005**
Percent (N) *All women*	34.1% (21)	24.3% (156)	0.189	28.0% (14)	11.9% (59)	**0.001**
Age groups						
18–34 years	21.4% (6)	30.5% (66)	0.474	41.2% (7)	19.5% (30)	0.034*
35+ years	39.4% (15)	23.0% (90)	**0.035**	23.3% (7)	8.0% (29)	0.001*
Place of residence						
Nuuk	36.4% (5)	21.4% (32)	0.256*			
Town	39.1% (11)	26.9% (80)	0.210			
Village	26.7% (5)	28.4% (44)	0.890*			
Total						
Percent (N) *Place of residence*						
Nuuk				44.8% (10)	17.7% (60)	**0.001**
Town				25.9% (45)	13.2% (171)	**0.001**
Village				7.0% (20)	9.2% (103)	0.631*

* Expected count less than 5.

Analysis of frequent use of marijuana according to place of residence was not stratified by gender because this resulted in too few individuals in each group. Greenland Inuit 2006–2010. Logistic regressions were stratified according to gender. Unadjusted and adjusted odds ratios (OR) with 95% confidence intervals (CI).

p≤0.05 values are present in bold.

### Frequent use of marijuana

Lifetime problem gambling was significantly associated with a frequent use of marijuana (Table II). Women displayed an odds ratio for frequent use of marijuana almost 3 times higher among lifetime problem gamblers compared to non-problem gamblers (both unadjusted and adjusted). The prevalence of frequent use of marijuana was significantly higher among lifetime problem gamblers in both age groups compared to non-problem gamblers. There was a significantly higher prevalence of frequent use of marijuana among lifetime problem gamblers compared to non-problem gamblers in the capital and towns, but not in the villages.

### Cross-addictive behaviours

**Fig. 2:**
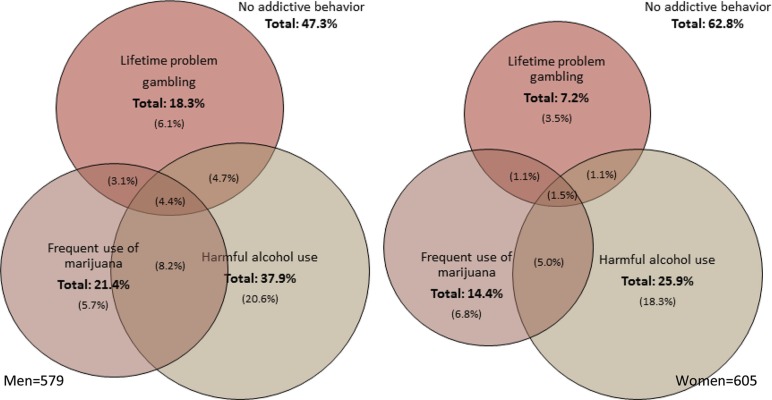
Prevalence of lifetime problem gambling, frequent use of marijuana in the past year, harmful alcohol use in the past year and the different combinations between the three as well as no addictive behaviour. The total prevalence of each of the three addictive behaviours as well as no addictive behaviour is written in bold figures. The prevalence of one addictive behaviour only, or the combination of two or three addictive behaviours, is written in (). Unadjusted percent, weighted for regional and age differences in the sample. Men (left), N=579, missing=404. Women (right), N=605, missing=601. Greenland Inuit 2006–2010.

In Fig. 2, the different combinations of lifetime problem gambling, harmful alcohol use and frequent use of marijuana were analysed for men and women (p<0.0001). A total of 8 different categories were constructed, that is 1) a category of no addictive behaviour, where neither lifetime problem gambling, frequent use of marijuana or harmful alcohol use were present; 2) lifetime problem gambling only; 3) harmful alcohol use only; 4) frequent use of marijuana only; 5) lifetime problem gambling combined with frequent use of marijuana; 6) lifetime problem gambling combined with harmful alcohol use; 7) frequent use of marijuana combined with harmful alcohol use; and, finally, 8) lifetime problem gambling combined with both frequent use of marijuana and harmful alcohol use.

Fifty three percent of men and 37% of women were either lifetime problem gamblers, used marijuana frequently, and/or had harmful alcohol use, or different combinations of the three. Harmful alcohol use was the most prevalent addictive behaviour among both men and women. Harmful alcohol use and frequent use of marijuana co-occurred with lifetime problem gambling among 12.2% of the men and 3.7% of the women. In comparison, harmful alcohol use and frequent use of marijuana co-occurred among 12.6% of men and 6.5% of women. Cross-addictive behaviour was found among 20.4% of men and 8.6% of women. For 4.4% of men and 1.5% of women all three addictive behaviours were present.

### Addictive behaviours in households with children

In total, the prevalence of one or more addictive behaviours in households with children was 44%. The prevalence of only one addictive behaviour in households with children was similar to households without children (32% vs. 28%), while the prevalence of two or more addictive behaviours was slightly higher in households without children compared to those with children (19% vs. 12%) (p=0.001, results not tabulated). The odds ratio for finding one or more addictive behaviours in a household was slightly lower in households with children compared to households without children (OR=0.76, CI 95%: 0.60–0.97; adjusted for age group and gender).

## Discussion

Harmful alcohol use and frequent use of marijuana were both associated with lifetime problem gambling and the prevalence of these addictive behaviours was significantly higher among lifetime problem gamblers compared to non-problem gamblers. Among problem gamblers the harmful use of alcohol increased with age while the opposite pattern characterised the non-problem gamblers. For lifetime problem gamblers, the gambling problems were more often than not combined with harmful alcohol use, a frequent use of marijuana or both – especially among men. More than half of the men and one third of the women displayed one or more addictive behaviours. This was also the case in almost half of the households with children.

Our findings regarding co-morbid behaviour are in agreement with international findings from other population-based studies. Lorains et al. (8) found a weighted mean estimate of 29.8% for alcohol use disorder among combined groups of problem and pathological gamblers across 3 population-based studies, although none of these were based on indigenous populations. Two of these studies used the original CAGE questionnaire and found a total prevalence for alcohol use disorder among problem gamblers of 14% in 2005 (24,25). Considering that the original version of CAGE measures harmful alcohol use in a lifetime perspective, while CAGE-C applies for the past year, the prevalence of harmful use of alcohol among lifetime problem gamblers in Greenland seems high – especially for men.

The combination of alcohol and marijuana in a cross-addictive behaviour is well-known among public health professionals in Greenland, but gambling problems have not systematically been included in strategies for prevention in this field. Our study shows, that the co-occurrence of harmful alcohol use and frequent use of marijuana with lifetime problem gambling is similar to the co-occurrence of harmful alcohol use and frequent use of marijuana. Problem gambling was measured in a lifetime perspective. This means that gambling problems may not be an issue any more, but despite this, there is a prevalent overlap with alcohol problems and frequent use of marijuana in the past year. Based on these observations, it seems important to consider gambling problems as a part of other addictive behaviours, even though these problems may not be current.

The high prevalence of one or more addictive behaviours in households with children suggests that many families and children in Greenland are affected by alcohol, gambling and marijuana. A report from the national program for early intervention in pregnant families in Greenland, which had 15% of all families with newborns from 2007–2010 enrolled, showed that all of these families were struggling with problems related to addictive behaviour (26). The negative influence of alcohol was also stated by the participants in the Inuit Health in Transition Greenland Survey, who rated alcohol to be the most important theme together with violence, when asked to rate the importance of the themes included in Greenland's first public health program “Inuuneritta 2007–2012”. This evaluation was further supported by results from the Survey of Living Conditions, where participants rated unemployment, alcohol and drug abuse, suicide, violence in the family and sexual abuse as serious social and health-related issues within the Greenlandic communities (27). An increase in social pathologies, such as addictive behaviours, is not only a challenge for Greenland, but a key indicator for indigenous people in the Arctic undergoing a similar transition (4). These issues need to be addressed through comprehensive public health strategies and preventive measures taking the complexity and the fundamental challenges related to sociocultural changes in the Greenlandic society into account.

Targeting interventions and treatment for pathological gamblers is a challenge because very few actually seek treatment for their disorder. A large US survey suggested that only 7–12% of the pathological gamblers seek treatment (28), and Kessler et al. (29) found that none of the problem gamblers in their study had ever received treatment for gambling problems, while half of them were treated for other mental disorders. This corresponds well with the experience from Greenland, where very few have been treated for pathological gambling. In consequence, there seems to be an important task for health professionals and social workers of screening for gambling problems when working with alcohol and substance use disorders.

Mental health disorders, such as bipolar disorder, major depression and antisocial personality disorders, have been found to be highly associated with problem gambling (8). However, mental health disorders could not be diagnosed on the basis of the health questionnaire in the Greenland Survey and, therefore, these were not included in the analyses, although they are likely to contribute to the prevalence of addictive behaviours. Given a seemingly high prevalence of mental disorders among Greenland Inuit (30,31), this association is important to consider when public health interventions and treatment are planned in practice. A study based on patients in the Greenlandic health care system found a prevalence of at least one ICD-10 diagnose among 49% of the patients, with anxiety, somatisation disorder, dysthymia and depression as the most prevalent diagnoses. However these results were based on a small population and cannot be generalised to the broad population. More knowledge about the prevalence and incidence of specific mental disorders among Greenland Inuit is needed and should take the possible link between mental disorders and problem gambling into consideration.

Future research should look into the aetiology of pathological gambling in relation to alcohol and substance use disorders, mental health disorders and their interrelatedness. Few studies have tried to establish what comes first based on retrospective questions asking participants to remember their age of onset for different addictive behaviours (29). Prospective studies are needed in order to ensure a better understanding of what comes first and how the prevention and treatment of one disorder might help to prevent other addictive behaviours. Furthermore, research should investigate the causes behind the increase in social pathologies such as addictive behaviours, which have been found to be a key feature in indigenous populations undergoing a similar transition as Greenland Inuit (4).

It is considered a strength of the study that different types of addictive behaviours are measured in the same survey. This has provided us with the opportunity to investigate cross-addictive behaviour among lifetime problem gamblers and the prevalence of different combinations of addictive behaviours. The number of problem gamblers in our sample is relatively large, which has made it possible to study the cross-addictive behaviours according to socio-demographic characteristics.

It is an important weakness of the study that the response to key variables followed a social gradient. When working with addictive behaviours and socially exposed groups, the missing data is expected to lead to an underestimated prevalence, because these groups are hard to reach through surveys. However, it should be noted that the prevalence found when combining the three different variables for addictive behaviours in this study is slightly higher, compared to the national prevalence estimates, when calculated separately. This is caused by the combination of missing data across the 3 measures, when they are combined in one variable, which reduces the “N”. Furthermore, it is possible that gambling and alcohol problems are under-reported in the study, due to the general stigma attached to addictive behaviours. Based on the existing data, we were not able to further asses a possible under-reporting regarding these subjects, but the number of missing data on the lie/bet screen was larger than the number of missing data on questions regarding different types of gambling (not included in this study). This suggests that people are more likely to answer questions about gambling activities, rather than gambling problems. Whether this has to do with participants not wanting to answer questions about gambling problems they might have, or with the fact that it is tempting to skip a couple of questions in a long questionnaire if they seem irrelevant to you personally, should be investigated through future qualitative methodological studies.

Finally, the lack of comparable measures for mental health disorders in the study may lead to a less comprehensive understanding of the prevalence and combinations of problem gambling, harmful use of alcohol and frequent use of marijuana.
